# Unraveling the crosstalk between extracellular ATP and glutamate in systemic ROS signaling

**DOI:** 10.1093/plphys/kiag241

**Published:** 2026-04-22

**Authors:** Hannah Rae Thomas

**Affiliations:** Assistant Features Editor, Plant Physiology, American Society of Plant Biologists, Rockville, Maryland, United States; Department of Horticulture, Zijingang Campus, Zhejiang University, 866 Yuhangtang Road, Hangzhou 310058, PR China

Wounding caused by biotic and abiotic factors is a common source of plant stress. When cells are damaged, various molecules are released into the apoplast. Some of these molecules, known as damage associated molecular patterns (DAMPs), act to trigger local and systemic wound responses. Extracellular ATP (eATP) and L-glutamic acid (Glu) are both present inside cells at high concentrations and when released into the apoplast during wounding,.

Extracellular ATP (eATP) and Glu bind to their apoplastic facing receptors, with eATP perceived by PURINORECEPTOR 2 KINASE 1 (P2K1) and P2K1, while Glu is bound to GLUTAMATE-LIKE RECEPTORS 3.3 (GLR3.3) and 3.6 (Choi et al. 2014; Mousavi et al. 2013; Pham et al. 2020). When Glu binds to GLR3.3 or GLR3.6, it directly triggers systemic Ca^2+^ influx, which, via calcium dependent protein kinases (CPKs), phosphorylates RESPIRATORY BURST OXIDASE HOMOLOGOUS PROTEIN D (RBOHD), thereby generating apoplastic reactive oxygen species (ROS) (Myers et al. 2022; Toyota et al. 2018). Therefore, Glu is required for both systemic Ca^2+^ and ROS signaling following wounding. In contrast, eATP activates P2K1 and P2K2, which can directly phosphorylate RBOHD, thereby generating systemic ROS (Chen et al. 2017). However, recent work has shown that eATP can activate the calcium CYCLIC NUCLEOTIDE-GATED CHANNELS, but the role of eATP in systemic Ca^2+^ signaling has not been shown (Sun et al. 2025). Previously, the authors found that both Glu and eATP could trigger similar systemic ROS waves, but the effects were not additive, suggesting that eATP and Glu ROS pathways may overlap (Myers et al. 2022).

Recently in *Plant Physiology*, Myers et al. (2026) set out to uncover the interaction between eATP and Glu-mediated systemic ROS. Using a whole-plant ROS and calcium imaging method, the authors explored how local and systemic responses occurred over time in *Arabidopsis thaliana.* First, the authors showed that eATP can indeed trigger systemic Ca^2+^ responses and that this depends not only on P2K1/2 but also on GLR3.3/3.6. Next, the authors showed that the same RBOHD phosphorylation site that was required for eATP-induced local ROS is also required for wound-induced systemic ROS.

To elucidate how the site of injury affects ROS signaling, the authors used a pin to create small wounds in vascular and nonvascular tissue. Based on this spatial wounding assay, they identified that P2K1/2 is required for nonvascular wound-induced ROS, but the *p2k1/2* double mutant could still propagate vascular ROS, whereas GLR3.3/3.6 was required for both vascular and nonvascular ROS (Fig. 1a). Interestingly, when wild type plants were wounded in both the nonvascular and vascular tissues, the ROS signals were additive, which suggests nonvascular and vascular wounds might activate distinct ROS pathways

**Figure 1:**
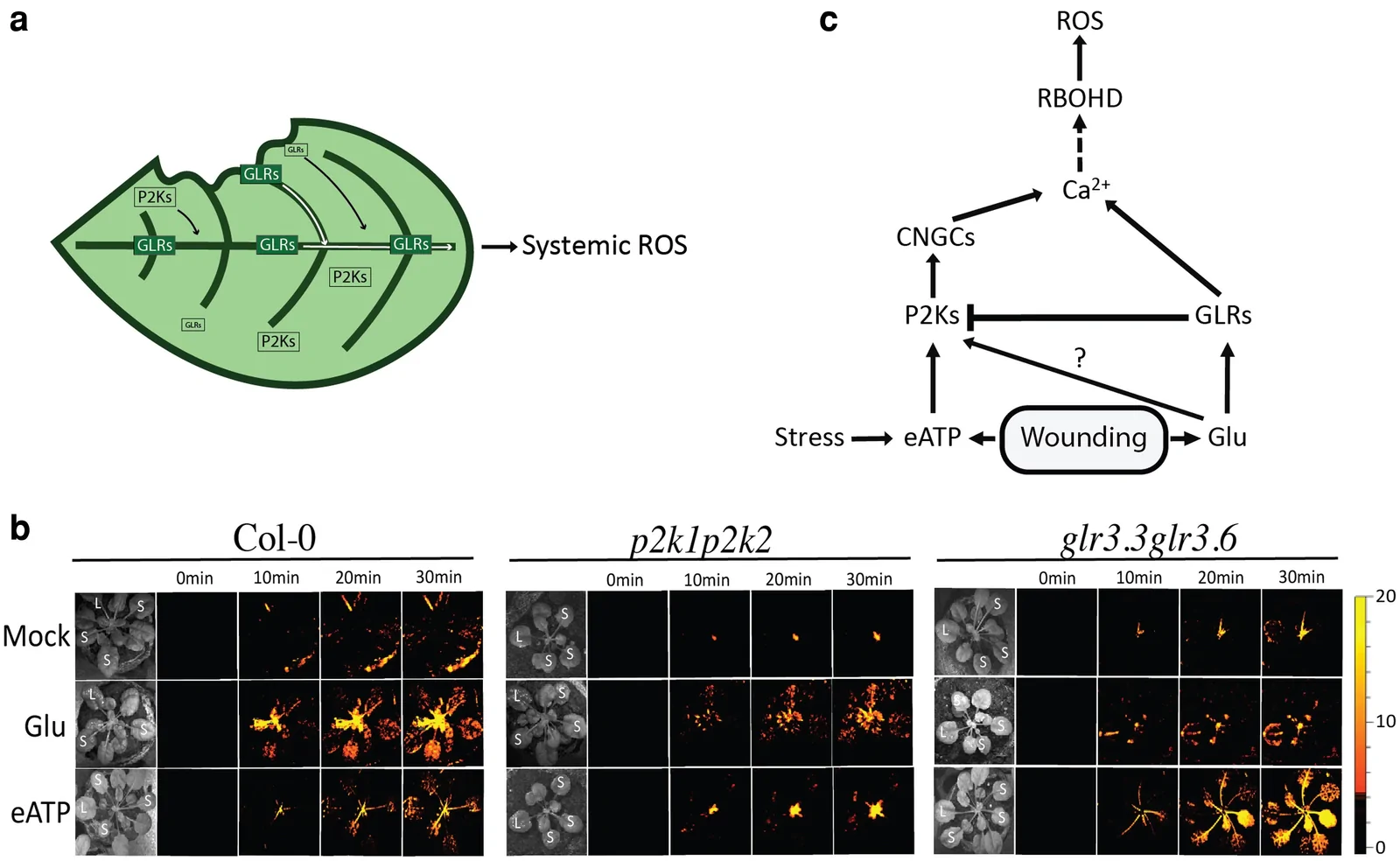
P2Ks and GLRs are required for DAMP-induced systemic ROS. (a) PURINORECEPTOR 2 KINASE 1 (P2K1) and P2K2 are required for nonvascular wound-induced systemic reactive oxygen species (ROS), while GLUTAMATE-LIKE RECEPTORS (GLRs) are required for nonvascular and vascular wound-induced systemic ROS. This is despite the vast majority of GLRs being expressed predominantly in vascular tissue. Vascular tissue is shown as dark green, and nonvascular tissue is shown as light green. The size of the receptor is shown to depict the main expression pattern. (b) Time lapse imaging of wild type (Col-0), *p2k1p2k2*, and *glr3.3glr3.6* plants treated with water (mock), glutamate (Glu) and extracellular ATP (eATP). In *p2k1p2k2*, eATP fails to elicit systemic ROS, while Glu treatment is reduced compared to Col-0. This suggests Glu signaling may be amplified by P2Ks. In contrast, *glr3.3glr3.6* fails to activate systemic ROS after Glu treatment, but displays greater eATP-mediated ROS than in Col-0, suggesting that GLRs repress eATP-mediated ROS. Treated leaves are noted with an “L” for local, while non-treated leaves are noted with an “S” for systemic. (c) A hypothesitical model of the interaction between eATP and Glu, where wounding triggers the release of eATP and Glu. Glu binds to GLRs following wounding, activating an influx of cytosolic calcium (Ca^2+^), which then activates RESPIRATORY BURST OXIDASE HOMOLOG D (RBOHD) to produce apoplastic ROS. In contrast, eATP binds to P2Ks following wounding of non-vascular tissue. P2Ks activate CYCLIC NUCLEOTIDE-GATED CHANNELS (CNGCs), which then trigger RBOHD-mediated ROS production. While Glu may utilize P2Ks as part of its Ca^2+^ response, GLRs repress eATP-induced ROS. In addition to wounding, heat stress can also induce eATP accumulation (Adapted from Myers et al., 2026).

The authors explored the interaction between Glu and eATP ROS signaling and showed that when the *p2k1p2k2* double mutant was treated with exogenous eATP, leaves lacked systemic ROS waves (Fig. 1b). The *p2k1p2k2* mutant also displayed a reduced Glu-mediated ROS response, suggesting that P2Ks may amplify Glu-mediated ROS. The *glr3.3glr3.6* double mutants showed no systemic ROS response to Glu but elicited a stronger ROS response to eATP treatment (Fig. 1b). These results showed that although eATP requires both P2Ks and GLRs for its systemic Ca^2+^ response, GLR3.3/3.6 appears to play a repressive role in eATP-induced ROS.

To identify which overlapping processes are activated by eATP and Glu treatments, the authors collected RNA-seq data from wild type and mutant samples. Despite both treatments triggering ROS, the 2 DAMPs showed very little overlap in differentially expressed genes. From the transcriptomics, the authors found that eATP treatment upregulated genes associated with heat stress, and by using heat treated seedlings the authors validated that high temperatures lead to the accumulation of eATP. In addition to wounding, the authors showed that eATP may also be released in response to additional abiotic stressors.

Overall, this work revealed that eATP and Glu have a complex relationship during wound responses that is sometimes synergistic and sometimes antagonistic (Fig. 1c), but several questions remain unanswered. For one, the authors note that GLR3.3/3.6 are predominantly expressed in vascular tissue and question why *glr3.3glr3.6* mutants would affect nonvascular ROS signals. However, it has been shown previously that GLR3.6 and, in particular, GLR3.3 are significantly involved in non vascular calcium wave propagation (Bellandi et al. 2022). This work highlights the divergent roles of calcium and ROS signaling, suggesting that GLRs in non vascular tissue function very differently in ROS propagation than in calcium signaling, but this hypothesis requires further investigation. Additionally, the authors note that the role of P2Ks in Glu signaling could be due to Glu-induced eATP rather than to Glu physically interacting with P2 K receptors, but they were unable to clarify this finding, and additional studies are required. Lastly, the complete interaction between the Glu and eATP signaling pathways remains unresolved. The authors suggest future studies on the roles of wounding, DAMP treatment, and expression location using a *p2k1/p2k2/glr3.3/glr3.6* quadruple mutant to fully elucidate these processes. While the precise regulation of DAMP-induced ROS signaling remains unclear, this work highlights the power of systemic signals to propagate defensive messages during infection and herbivory, and the importance of whole plant imaging as a powerful tool in plant molecular biology.

## Recent related articles in *Plant Physiology*

Arkadipta Bakshi, Simon Gilroy, Calcium signaling in hypoxic response, *Plant Physiology*, 197, 1, January 2025, kiae654, https://doi.org/10.1093/plphys/kiae654

Yosef Fichman, María Ángeles Peláez-Vico, Asha Kaluwella Mudalige, Hyun-Oh Lee, Ron Mittler, So-Yon Park, Rapid plant-to-plant systemic signaling via a *Cuscuta* bridge, *Plant Physiology*, 196, 2, October 2024, Pages 716–721, https://doi.org/10.1093/plphys/kiae339

## Data Availability

Not applicable

## References

[kiag241-B1] Bellandi A et al 2022. Diffusion and bulk flow of amino acids mediate calcium waves in plants. Sci Adv. 8:eabo6693. 10.1126/sciadv.abo6693. 36269836 PMC9586480

[kiag241-B2] Chen D et al 2017. Extracellular ATP elicits DORN1-mediated RBOHD phosphorylation to regulate stomatal aperture. Nat Commun. 8:2265. 10.1038/s41467-017-02340-3. 29273780 PMC5741621

[kiag241-B3] Choi J et al 2014. Identification of a plant receptor for extracellular ATP. Science. 343:290–294. 10.1126/science.343.6168.290. 24436418

[kiag241-B4] Mousavi SAR, Chauvin A, Pascaud F, Kellenberger S, Farmer EE. 2013. GLUTAMATE RECEPTOR-LIKE genes mediate leaf-to-leaf wound signalling. Nature. 500:422–426. 10.1038/nature12478. 23969459

[kiag241-B5] Myers RJ, Fichman Y, Stacey G, Mittler R. 2022. Extracellular ATP plays an important role in systemic wound response activation. Plant Physiol. 189:1314–1325. 10.1093/plphys/kiac148. 35348752 PMC9237675

[kiag241-B18] Myers RJ et al. 2026. L-Glutamic acid negatively regulates extracellular ATP-induced reactive oxygen species signaling, Plant Physiol. 201:kiag230. 10.1093/plphys/kiag230. 42051146 PMC13273574

[kiag241-B6] Pham AQ, Cho SH, Nguyen CT, Stacey G. 2020. Arabidopsis lectin receptor kinase P2K2 is a second plant receptor for extracellular ATP and contributes to innate immunity. Plant Physiol. 183:1364–1375. 10.1104/pp.19.01265. 32345768 PMC7333714

[kiag241-B7] Sun Y et al 2025. Activation of the CNGC2-CNGC4 channel complex by P2K1-mediated phosphorylation links extracellular ATP perception to calcium signaling in plant immunity. Mol Plant. 18:1130–1142. 10.1016/j.molp.2025.06.001. 40474480

[kiag241-B8] Toyota M et al 2018. Glutamate triggers long-distance, calcium-based plant defense signaling. Science. 361:1112–1115. 10.1126/science.aat7744. 30213912

